# Lengthening of free fibular grafts for reconstruction of the residual leg length discrepancy

**DOI:** 10.1186/s12891-019-2445-z

**Published:** 2019-02-08

**Authors:** Xianghong Zhang, Tingting Zhang, Tang Liu, Zhihong Li, Xiangsheng Zhang

**Affiliations:** 1 Department of Orthopedics, The Second Xiangya Hospital of Central South University, 139# Middle Renmin Road, Changsha, Hunan 410011 People’s Republic of China; 2 Department of Orthopedics, Liuzhou General Hospital, Guangxi University of Science and Technology, Liuzhou, 545006 Guangxi China; 3 Department of Obstetrics and Gynecology, The Second Xiangya Hospital, Central South University, Changsha, 410011 Hunan China

**Keywords:** Vascularized free fibular graft, Bone lengthening, Osteomyelitis, Unilateral external fixator

## Abstract

**Background:**

We evaluated our results of lengthening of free vascularized fibular grafts using a unilateral external fixator in patients with residual leg length discrepancy after free vascularized fibular graft for lower limb reconstruction.

**Cases presentation:**

Two patients were administrated to our hospital with residual tibial length discrepancy after vascularized free fibular graft surgery. Lengthening of the free vascularized fibular graft with a unilateral external fixator was performed to correct the leg length discrepancy. Both patients recovered well with no difficult in activities of daily living at the last follow-up.

**Conclusions:**

This study shows that lengthening of free vascularized fibular grafts with an external fixator is an effective treatment for massive residual leg shortening after vascularized free fibular graft surgery.

## Background

Massive segmental bone defects often arise from tumor resection, congenital malformation, trauma, osteomyelitis, and so on [1–3]. Though bone micro-vascular transfer has been proven to be an effective technique for reconstruction of larger bone defects [4], the management of larger bone defects remains a considerable surgical challenge [5–7]. Massive structural allografts and vascularized fibular autografts have their own limitations, such as lack of sufficient transplantable materials, donor site morbidity, inflammation, and resorption of the grafts [8, 9]. Since described by Taylor in 1975 [10], the free vascularized fibular grafts (FVFG) has been widely used for reconstructing a segmental bone defect [11–15]. However, there are few reports on lengthening of FVFG for reconstruction the residual leg length discrepancy (LLD). In this study, we evaluated the results of lengthening of FVFG using a unilateral external fixator [16, 17] in 2 patients with residual tibial length discrepancy after FVFG for lower limb reconstruction.

## Case presentation

### Case 1

**Fig. 1:**
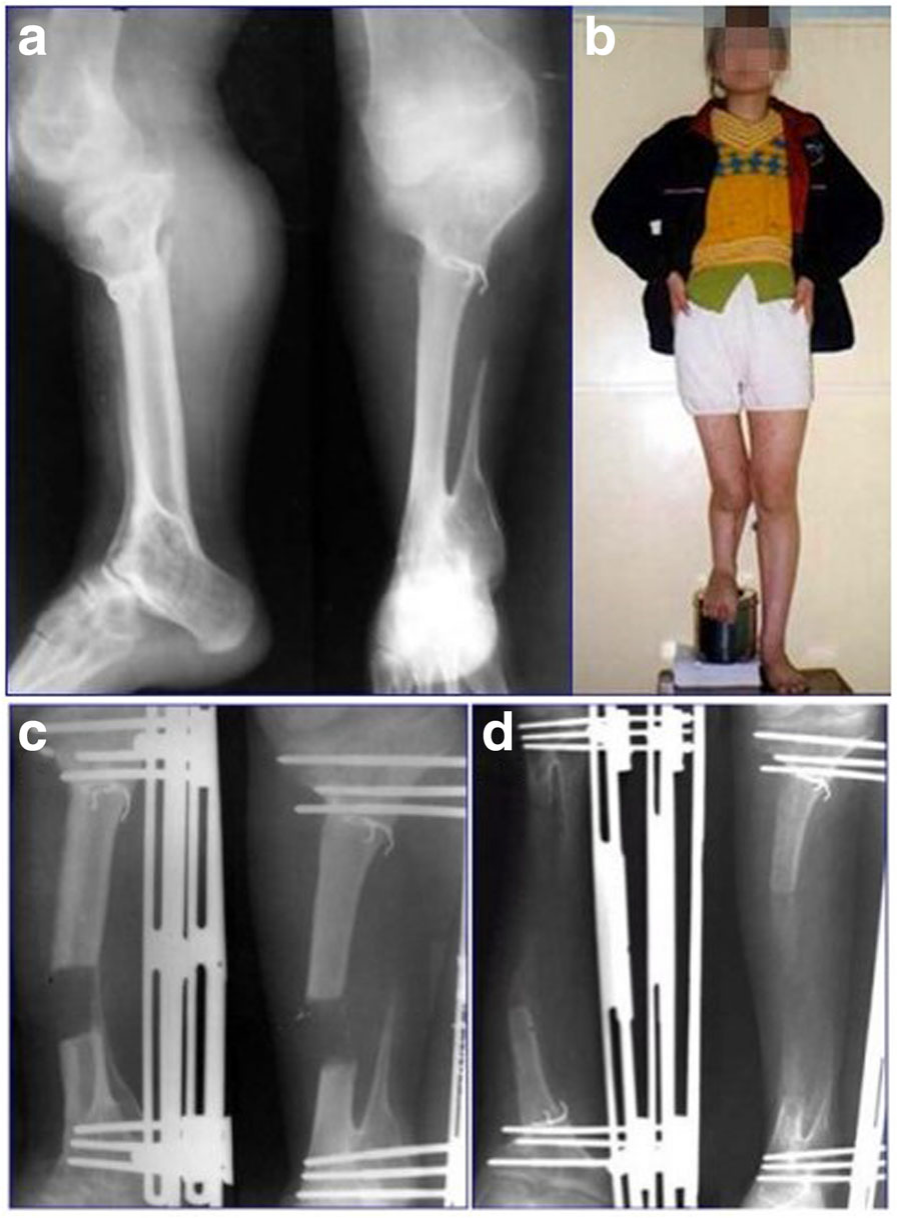
The X-ray film of case 1 reveals reconstruction of the tibia with a FVFG was done (**a**), she has 18 cm shortness at the right limb (**b**), the length correction program with unilateral external fixator was done and the callus regenerated well (**c**, **d**)

**Fig. 2:**
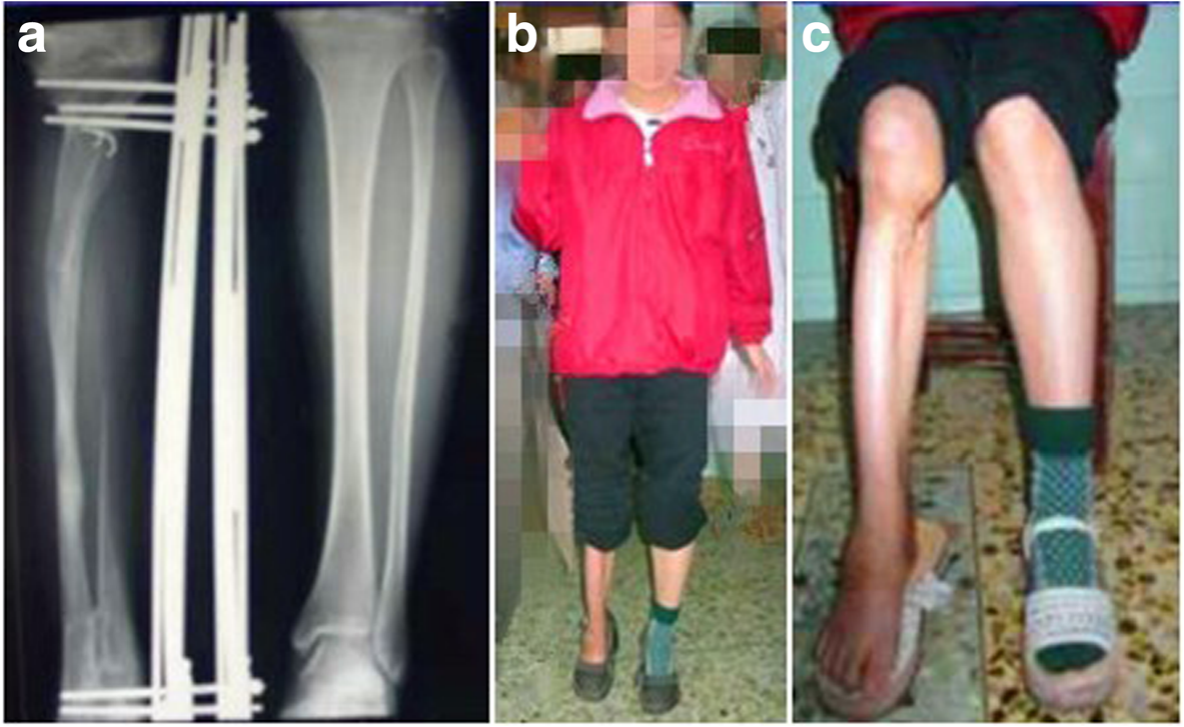
The X-ray film reveals a total lengthening of 18.0 cm was achieved (**a**), and she can walk well without walking aids or braces (**b**), and the knee and ankle’ function were near normal at the last follow-up (**c**)

A 16-year-old female patient was first administrated to our hospital because her right leg was 18 cm shorter than contralateral side (Fig. 1). When she was 4-year-old, she contracted right tibia pyogenic osteomyelitis, leading to a 6-cm tibial absorption involving the distal epiphysis. The patient had no other past medical history. When she was 5, the tibia defect was reconstructed by her ipsilateral vascularized fibular graft. However, progressive leg length discrepancy developed with limited range of joint motion (ROM) (Table 1). When she was 16, lengthening of FVFG with a unilateral external fixator was performed to correct the LLD (Fig. 1). We executed an open osteotomy at the level of the middle metaphysis of the matured fibular graft. The patient received preventive intravenous antibiotic (Cefuroxime) for 72 h. The latency period was 7 days after the operation and distraction was performed at a rate of 1.0 mm per 36 h in four increments of 0.25 mm, and when the length of bone regeneration had reached approximately 6.0 cm, the distraction rate was reduced to 1.0 mm every 48 h [18]. Clinical and radiological examination was carried out every 15 days to assess new bone formation and the pin sites [18]. The rate was adjusted according to the discomfort and swelling of the limb and the quality of the regenerate bone like our previous study [18]. Partial weight-bearing was allowed as soon as union of the vascularized fibula graft on either junction was observed on radiographs. We achieved equalization with a unilateral external fixator in 26.5 months. The mean external fixation index was 44.2 day/cm. She had a pin-track infection and local inflammation, which were managed with pin care and oral antibiotics. The unilateral external fixator was removed when at least three of the four cortices were observed to be united on anteroposterior and lateral radiographs. She was able to walk without walking aids or braces, and to perform almost all activities of daily living with no difficulty based on the recommended criteria [19]. The results were divide into bone results and functional results. Based on the criteria recommended by Paley et al. [20, 21], bone result were excellent, and function result were good (Fig. 2, Table 1).

**Table 1:** Range of motion before and after the lengthening surgery

	Extension/Flexion of the knee		Plantar flexion/dorsiflexion of ankle	
	Pre-operation	After the operation	Pre-operation	After the operation
Case 1	0–0/110°	0–0/95°	0–18/15°	0–13/8°
Case 2	0–0/108°	0–0/92°	0–15/10°	10–13/−10°

### Case 2

**Fig. 3:**
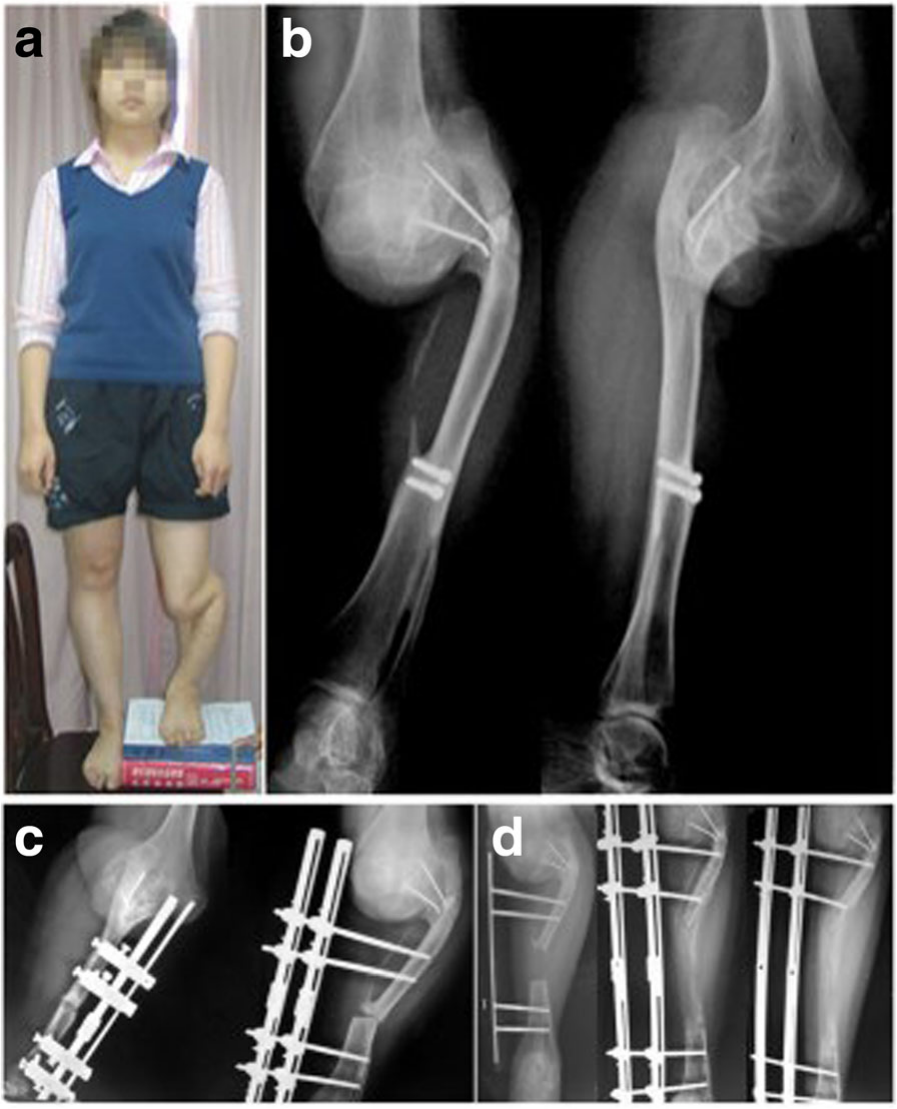
This clinical image of the case 2 shows 9 cm shortness at the left limb (**a**), and the preoperative X-ray film reveals her upper and most middle left tibia involving the proximal epiphysis were fusioned together with fibula (**b**), and the length correction program with unilateral external fixator was done and the callus regenerated well (**c**, **d**)

**Fig. 4:**
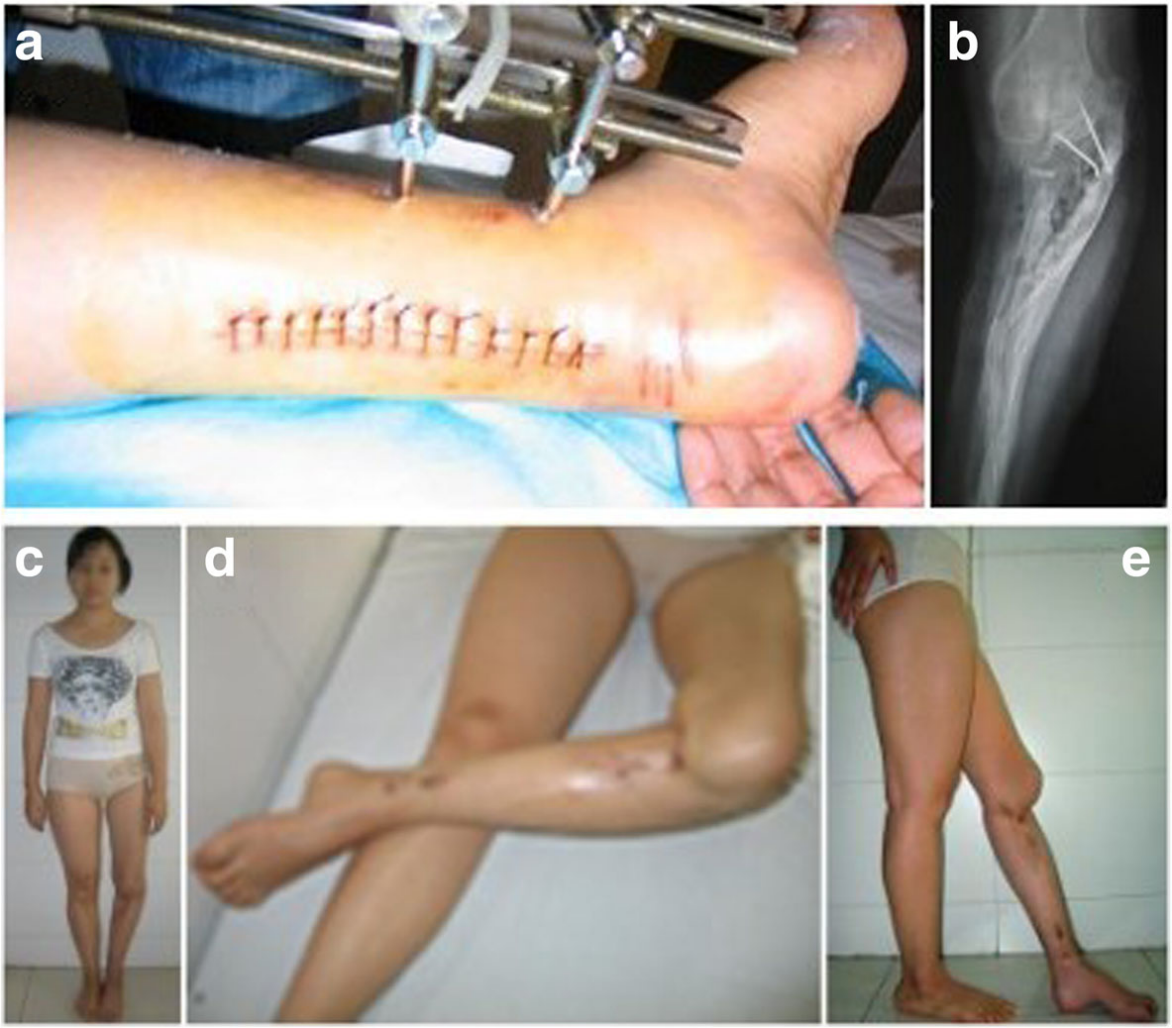
This image shows a surgery of achilles tendon lengthening was performed to correct the relapse clubfoot deformity (**a**), and a surgery of bone grafts under the left tibial plateau was performed (**b**), and she can stand on straightly without walking aids or braces (**c**, **d**, **e**)

A 17-year-old female patient was administrated to our hospital because her left leg was 9 cm shorter than contralateral side (Fig. 3). When she was 6 years old, she was diagnosed as left tibia pyogenic osteomyelitis. She had no other past medical history. Her upper and almost the middle left tibia were absorbed. She received ipsilateral vascularized fibular graft to reconstruct bone defect at 8-year-old. The distal tibia was fused with fibula. Subsequently, progressive LLD developed. To correct the discrepancy, gradual length correction with a unilateral external fixator was started after being administrated to our hospital (Fig. 3). The lengthening procedure was started at 9 years after fibular graft surgery. A lateral incision was used in the fibula for osteotomy. In order to sustain the anatomic axis, each set of pins was positioned in the same plane and perpendicular to the long axis of the proper lower limb alignment. The patient received preventive intravenous antibiotic (Cefuroxime) for 72 h. The latency period was 7 days after the operation and the rate of distraction was like case 1 according to our previous study [18]. Time to bone union was expressed in every 15 days and it was determined using the follow-up x-rays. Physiotherapy, daily nursing care, and regular follow-up were performed together with distraction. We achieved symmetry with a unilateral external fixator in 13.5 months. The mean external fixation index was 45.0 day/cm. The patient had a relapse clubfoot deformity gait when symmetry was achieved (Table 1). Achilles tendon lengthening surgery was performed to correct the relapse clubfoot deformity (Fig. 4). Partial weight-bearing was allowed as soon as union of the vascularized fibula graft on either junction was observed on radiographs. She can’t full weight-bearing until complete union of the vascularized fibula and massive bone allograft to host bone was evident. The results were divide into bone results and functional results. Based on the criteria recommended by Paley et al. [20, 21], bone result and function results were good. (Fig. 4, Table 1).

## Discussion and conclusions

The surgical management of large bone defect is challenging for orthopedic surgeons. Several different treatment options for this complex disease, such as autografts, allografts, induced membranes, microvascular osseous transfer, as well as bone transport [15, 19, 22–24]. Free fibular grafting is considered as one of the standard salvage procedures for reconstructing segmental skeletal defect [25, 26]. Using free vascularized bone grafts generates better results than using non-vascularized bone grafts [27]. A vascularized osseous transfer has several advantages during the early process of bone repair, including an enhanced union capacity, the potential for subsequent osseous hypertrophy and greater strength [28].

Though FVFG is a reliable method for bone defect, it is also associated with a high complication rate (Table 2). The most common complications reported were mechanical failure, graft fracture, nonunion, infection, and associated complications at the donor site [29–31]. Although a number of studies have documented the complications that occur during the process of bone repair, little has been reported about the treatment of late complications, such as residual LLD. The complication of residual LLD has not been reported frequently, and a lengthening procedure after FVFG has rarely been reported. There are no guidelines for the lengthening procedure of a FVFG and various types of fixators have been used for bone lengthening. Some authors suggest that fibular graft lengthening using an external fixator is a safe and effective method for correcting LLD [32].

**Table 2:** Summary of published series

Study	Cases	Topography	Indication	LLD	The site of osteotomy	The interval between FVFG and lengthening (years)	Lengthening procedure after LVFG	Rhythm of lengthening	Complications
Ilizarov S et al. [12]	1	Humerus	Tumor resection	9	The widest area of free fibul	9	With monolateral frame	Start on 0.25 mm three times per day for 10 days, then changed to 0.25 mm two times per day	Well-remodeled regenerate and no bone resoption, Right elbow ROM was 0°-150°extebsion-flexion.
Kanaya F et al. [15]	1	Tibia	CPT	6.2	Do not state	8	With Ilizarov Fixator	Do not state	Rugular activity.
	1			4		7			Baseball, run.
	1			4.5		9			Rugular activity.
Courvoisier A et al. [32]	1	Tibia	Tumor resection	3	Through the FVFG	15	With Ilizarov Fixator	At a 1-mm per day	No complications occurred during lengthening, had normal gait and normal knee and ankle range of motion.
	1	Tibia	Tumor resection	10	Proximal to the FVFG	7	With a monolateral external frame	At a 1-mm per day	Full weightbearing, normal gait, a knee flextion contracture of 10°, and the ankle was stiff with residual equinus of 20°.
	1	Tibia	CPT	11	The tibia above the FVFG	10	With Ilizarov Fixator	At a 1-mm per day	The knee was stable with a flexion contracture of 10° and maximal flexion range of 140°, and the hind foot had a resudual valgus of 10°and equinus of 20°.
Jupiter JB et al. [28]	1	Tibia	CPT	8	Through the FVFG	12	With Ilizarov Fixator	Do not state	The osteotomy sites had healed, and the patient can bear full weight.
Shaw KJ et al. [35]	1	Humerus	Tumor resection	8	Do not state	Do not state	With a monoaxial external fixator	Do not state	A 20° elbow flexion contracture persisits.
Our cases	1	Tibia	Osteomyelit	18	The widest area of free fibul	11	With a unilateral external fixator	Distraction at the same as that for native bone	The mean external fixation index has no significant difference to native normal bone,Her ROM of knee and ankle was shown in this content.
	1	Tibia	Osteomyelit	9	The widest area of fibul	9	With a unilateral external fixator	Distractionat the same as that for native bone	Has a relapse clubfoot deformity gai, the ROM of knee and ankle was shown in this content.

The mechanical performance of vascularized fibular transfer-based lower-limb reconstruction is dependent on the ability of the graft to hypertrophy. FVFG hypertrophy has been well studied. El-Gammal et al. conducted a study on 25 patients with lower limb tumors who underwent reconstruction with vascularized fibula graft, and suggested that hypertrophy of the vascularized fibular graft is a time-related phenomenon and generally affected by the age of the patient [33]. However, the cause of hypertrophy remains unclear. Fibular graft hypertrophy evaluated with standard radiographs is a prerequisite for distraction.

Based on the criteria recommended by Paley et al. [20, 21], the results were divided into bone results and functional results. For bone results, four criteria were evaluated: union; infection; deformity and leg-length discrepancy. An excellent bone result was one in which there was union, no infection, deformity< 7°, and leg-length discrepancy < 2.5 cm, and an good result was union, plus two of the other criteria. For functional results, five criteria were recommended, including pain; need for walking aids or braces; foot, ankle, or knee deformity or contracture; ankle and/or subtalar loss of range of motion as compared with the preoperative range; and ability to return to normal activities of daily living and/or work. Our two cases could walk well without support at last follow-up. Based on the above criteria, functional results were good due to ankle loss of range of motion as compared with the preoperative range.

The optimal time to perform lengthening on a transferred bone is also a main issue that should be taken into consideration by the orthopedic surgeon (Table 2). De Boer and Wood [34] suggested that 80% hypertrophy of the FVFG should be observed at the 2-year’ follow-up. Courvoisier A et al. reported that three years after the FVFG seems to be a safe delay [32]. In their study, the mean age of their patients at surgery was 12 years, and the mean interval between the end of reconstruction and lengthening was 10 years. Jupiter et al. evaluated the results of skeletal reconstruction performed through a mature, vascularized fibular graft in five patients [28]. In their study, the secondary reconstruction was successful in all five patients, and the average time interval between the original transplant and the secondary reconstruction was 68 months [28]. Ilizarov S et al. reported a patient with resection of humerus for osteosarcoma, and initial reconstruction of the bone defect with FVFG. Then, the FVFG was subsequent lengthened four years later [12]. They noted that the optimal time for children to do a secondary lengthening was their growth plate closure, so that the lengthening goal is clear. Shaw et al., described a patient with humerus osteosarcoma who underwent limb salvage surgical resection with a vascularized fibula graft followed by limb lengthening [35]. They suggested that the lengthening procedure should be performed once the reconstruction of the extremity with the vascularized fibular autograft is stable. The interval between the end of reconstruction and lengthening in our first patient was 11 years, and this interval in our other patient was 9 years.

There are no guidelines for the lengthening of a FVFG. We adopted the same lengthening protocol as that for native bone [36]. In order to achieve the desired distraction without joint instability, the knee and foot were not included in the frame and daily physiotherapy was required. No matter where you make the osteotomy, the literature [32] showed that bone ingrowth were always succeed (Table 2). The site of osteotomy was performed in the FVFG in our two cases, and bone ingrowth were achieved at last. Ilizarov et al. recommend a rate of distraction of 0.5 mm per day or less to sustain good formation [12]. However, the rhythm of distraction in our cases was performed at the same rate as that of native bone. No specific complications occurred during the lengthening procedure in our patients and the mean external fixation index also showed no significant differences to the lengthening of other bone distraction with a unilateral external fixator. However, it is still difficult to provide formal guidelines for the lengthening procedures after a FVFG due to a lack of cases. Therefore, further studies should be performed and orthopedic surgeons should highlight their key points.

In conclusion, our study shows that lengthening of FVFG with an external fixator is an effective treatment for massive residual leg shortening after vascularized free fibular graft for lower limb reconstruction.

## Abbreviations

FVFG: Free vascularized fibular grafting
LLD: Limb-length discrepancy
ROM: Range of joint motion
